# Complete Coding Sequences of Six Toscana Virus Strains Isolated from Human Patients in France

**DOI:** 10.1128/genomeA.00454-16

**Published:** 2016-05-26

**Authors:** Amal Baklouti, Isabelle Leparc-Goffart, Geraldine Piorkowski, Bruno Coutard, Nicolas Papageorgiou, Xavier De Lamballerie, Rémi N. Charrel

**Affiliations:** a UMR Emergence des Pathologies Virales (EPV: Aix-Marseille University - IRD 190 - Inserm 1207 - EHESP), Marseille, France; b Fondation IHU Méditerranée Infection, APHM Public Hospitals of Marseille, Marseille, France; c Institut de Recherche Biomédicale des Armées, National Reference Laboratory for Arboviruses, Marseille, France; d CNRS, AFMB UMR 7257, Marseille, France

## Abstract

Toscana virus (TOSV) is an arthropod-borne phlebovirus belonging to the *Sandfly fever Naples virus* species (genus *Phlebovirus*, family *Bunyaviridae*). Here, we report the complete coding sequences of six TOSV strains isolated from human patients having acquired the infection in southeastern France during a 12-year period.

## GENOME ANNOUNCEMENT

Toscana virus (TOSV) belongs to the *Sandfly fever Naples virus* species within the genus *Phlebovirus* in the family *Bunyaviridae*. It is transmitted by phlebotomine sandflies belonging to the genus *Phlebotomus* in the Old World. TOSV is an enveloped virus with a single-strand negative-sense RNA genome consisting of three segments (large [L], medium [M],and small [S]). L encodes the virus polymerase, M encodes Gn and Gc, the two glycoproteins, and the nonstructural proteins NSm, and S encodes the nonstructural proteins NSs and the nucleoprotein (N) (1).

The phylogenetic relationships indicate that TOSV strains are subdivided into 3 lineages (A, B, and C) that are more or less congruent with the geography. Lineage A strains have been identified in Italy, France, Algeria, Tunisia, and Turkey (2–5); lineage B strains were detected in Spain, Portugal, France, Morocco, and Turkey (6, 7); lineage C was detected on a molecular basis only (no virus isolation) in Croatia and Greece. Interestingly, lineages A and B cocirculate in France and Turkey (8, 9).

Despite the large geographic area where human populations are exposed to TOSV, only 10 full-length sequences were available at the outset of this study, three for lineage A and seven for lineage B; of these 10 sequences, seven originated from sandflies and three from humans. We determined the complete coding sequence of five lineage B TOSV (TOS-4906, TOSV-11368, TOSV-9028, TOSV-54441132704, and TOSV-5963930705) and one lineage A (TOSV-5904242804) strain. This study brings the total to 6 complete sequences of TOSV that are publicly available.

All strains were isolated in Vero cells from clinical samples collected in human patients. Viral RNA extracted from an infected Vero cell culture supernatant at passage 3 was used for next-generation sequencing (NGS) using PGM Ion Torrent (Life Technologies) after nonspecific amplification. Reads were mapped on TOSV reference sequences to produce long consensus contigs. The criteria for read validation were >50% mapping in length and >80% identity to the selected reference sequence ISS.Phl.3 (GenBank accession numbers NC_006318 to NC_006320) for lineage A TOSV-5904242804; for the other 5 lineage B strains, sequences were mapped onto TOSV-AR 2007 (GenBank accession numbers EF656361 to EF656363).

The open reading frames (ORFs) of the 6 strains encoded N (254 amino acids [aa]), NSs (316 aa), Gn (527 aa), Gc (502 aa) and L (2,101 aa). The full-length sequences of each gene were aligned with homologous TOSV sequences using the CLUSTAL algorithm. Neighbor-joining analysis (Kimura 2-parameter and *p*-distance models) was performed by MEGA6, with 1,000 bootstrap pseudoreplications. These six TOSV strains are available for academic research purposes in the collection of the European Virus Archive (EVAg).

### Nucleotide sequence accession numbers.

The sequences of TOSV-9028, TOSV-4906, TOSV-11368, TOSV-5963930705, TOSV-5904242804, and TOSV-54441132704 have been deposited in GenBank under the accession numbers KU904263 to KU904265, KU922125 to KU922127, KU925897 to KU925899, KU935733 to KU935735, KU935736 to KU933538, and KX010932 to KX010934.
